# STED lithography in microfluidics for 3D thrombocyte aggregation testing

**DOI:** 10.1186/s12951-020-00762-8

**Published:** 2021-01-18

**Authors:** Bianca Buchegger, Alexander Tanzer, Sandra Posch, Christian Gabriel, Thomas A. Klar, Jaroslaw Jacak

**Affiliations:** 1 Institute of Applied Physics and Linz Institute of Technology (LIT), Johannes Kepler University Linz, Altenberger Straße 69, 4040 Linz, Austria; 2 University of Applied Sciences, Upper Austria School of Medical Engineering and Applied Social Sciences, Garnisonstraße 21, 4020 Linz, Austria; 3 Institute of Biophysics, Johannes Kepler University Linz, Gruberstraße 40, 4020 Linz, Austria; 4 Ludwig Boltzmann Institute for Experimental and Clinical Traumatology, Donaueschingenstraße 13, 1200 Vienna, Austria

**Keywords:** Multiphoton polymerization lithography, Stimulated emission depletion lithography, Microfluidics, Thrombocyte activation, Von Willebrand factor

## Abstract

Three-dimensional photopolymerization techniques such as multiphoton polymerization lithography (MPL) and stimulated emission depletion (STED) lithography are powerful tools for fabricating structures in the sub-µm range. Combining these techniques with microfluidics enables us to broaden the range of their applications. In this study, we show a microfluidic device enhanced with MPL structures carrying STED-lithographically written nanoanchors that promote binding of the von Willebrand factor (vWF). The density of vWF is adjusted by varying the number of the nanoanchors on the 3D structures. This allows us to study the impact of the density of vWF on the activation of thrombocytes. The activation of the thrombocytes seems to decrease with the density of vWF on the 3D scaffolds inside the microfluidic channels.
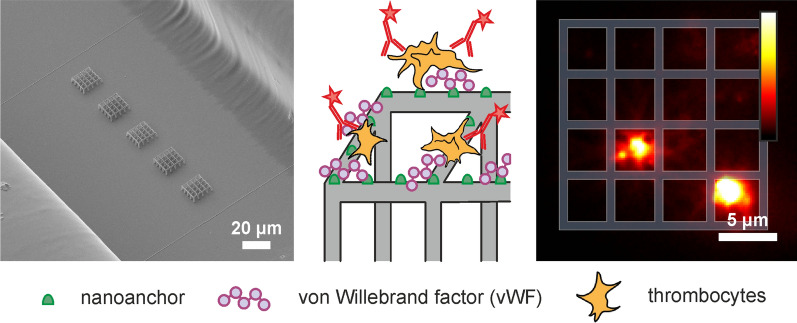

## Introduction

Multiphoton polymerization lithography (MPL) is a powerful technology that allows for 3D fabrication of complex structures in arbitrary shapes with sub-micrometer resolution [1]. Currently, MPL has numerous applications in microfluidics, biotechnology, and cell biology as well as in the fields of metamaterials and plasmonics [2–6]. In MPL, a photoresist is exposed to femtosecond laser pulses to induce polymerization by multiphoton absorption. It has become a versatile technique for the fabrication of full 3D structures with lateral sizes of 100 nm and axial dimensions below 300 nm (for infrared excitation) [1, 7]. Similar to sub-diffractional fluorescence microscopy, the lithographic resolution can be enhanced by stimulated emission depletion (STED) [8, 9] or related techniques [10–12]. STED lithography significantly reduces the feature size of MPL written structures and increases the achievable resolution [9, 13–15].

The versatility in sample preparation, the achieved feature sizes and resolution, and the flexibility in geometry makes MPL very well suited for direct writing into microfluidic channels, even under flow conditions [16–18]. Until now, microfluidic systems enhanced with sub-µm 3D lithography structures have been used for lab-on-chip devices, filters, mixers etc. [19–23]. Furthermore, microfluidics containing three-dimensional micro- and nanostructures have been used to control the microfluidic channel geometry and the flow dynamics [24, 25]. Modifications of the flow (e.g. blood flow) have been introduced by writing movable micromachines using MPL [26, 27]. Structured microfluidic channels have been used in numerous biotechnological applications and have demonstrated their ability to modify biological systems, including influencing cell growth; proliferation; and activation [28–31]. Microfluidics is frequently used to mimic blood circulation systems. By the use of microfluidics, it has been shown that perturbations in the flow can induce very significant impacts on cellular processes including thrombocyte activation [32, 33]. More recently, microfluidics enhanced with 3D lithography structures have been used to provide a biomimetic and biohybrid model of the blood-tissue barrier [34]. However, STED-enhanced lithography within microfluidics for biological studies has not yet been utilized.

In this publication, we show that STED enhanced structures inside of microfluidics can be used to stimulate cellular processes. Specifically, the small feature sizes that can be written with STED-lithography allow a great flexibility regarding protein density on the polymeric nanostructures, even down to a single molecule level. Hereby, we use multimaterial 3D structuring, where one component (a protein-repellent scaffold) is written by MPL and nanoanchors for proteins are added by STED-lithography. Such a strategy is based on multimaterial MPL which has been used (however without STED), for instance, for selective chemical functionalization [35, 36] for controlled 3D cell scaffolds [37–39], and for microactuators [40]. It may even go up to 3D structures composed out of five different materials, written within flow cells [41]. Also in the case of STED lithography, there is an advantage to work with two different resists: In most cases, sub-diffractional nanostructuring, which is highly precise but intrinsically slow, is needed only in some areas of the structure. Those will be written with a resist (specifically with a photostarter) optimized for STED. The rest of the structure does not require high precision and can hence be written with a formulation (in particular with a photostarter) that is optimized for writing speed.

In our case, the MPL fabricated structures, used as 3D substrates for the finer, STED enhanced features, consist of a protein-repelling polymer. Sub-100 nm small nanoanchors on top of the MPL structures were written with STED lithography, using a protein adhesive polymer [42–44]. The nanoanchors promote binding of the von Willebrand factor (vWF), a protein, which binds to the vWF receptor (Glycoprotein Ib/V/IX) in the thrombocyte membrane in order to mediate hemostasis. Using single molecule fluorescence microscopy, we were able to determine the number of vWF molecules anchored onto each STED lithographically derived nanostructure, which was three vWF molecules on average. Such tight control over small numbers of attached proteins is only possible using STED-lithography, but not with MPL. Under flow conditions, the structures were incubated with thrombocytes (platelet concentrate). We were able to show that the artificially created 3D environment with a defined number of vWF molecules impacts the activation of the thrombocytes.

To reveal thrombocyte activation, we monitored the abundance of the protein CD62p (p-selectin). Upon activation, alpha granule are fused with the thrombocyte membrane; hence CD62p is included into the membrane helping to immobilize thrombocytes [45]. The activation was monitored using an anti-CD62p antibody conjugated to Alexa^®^647 fluorophore. For the analysis, the overall signal of activated thrombocytes on top of the structures was measured. Interestingly, the analysis revealed that the overall activation signal correlates inversely with the number of nanostructures carrying vWF. A smaller density of vWF induces a stronger thrombocyte activation.

## Materials and methods

### Microfluidic device

**Fig. 1:**
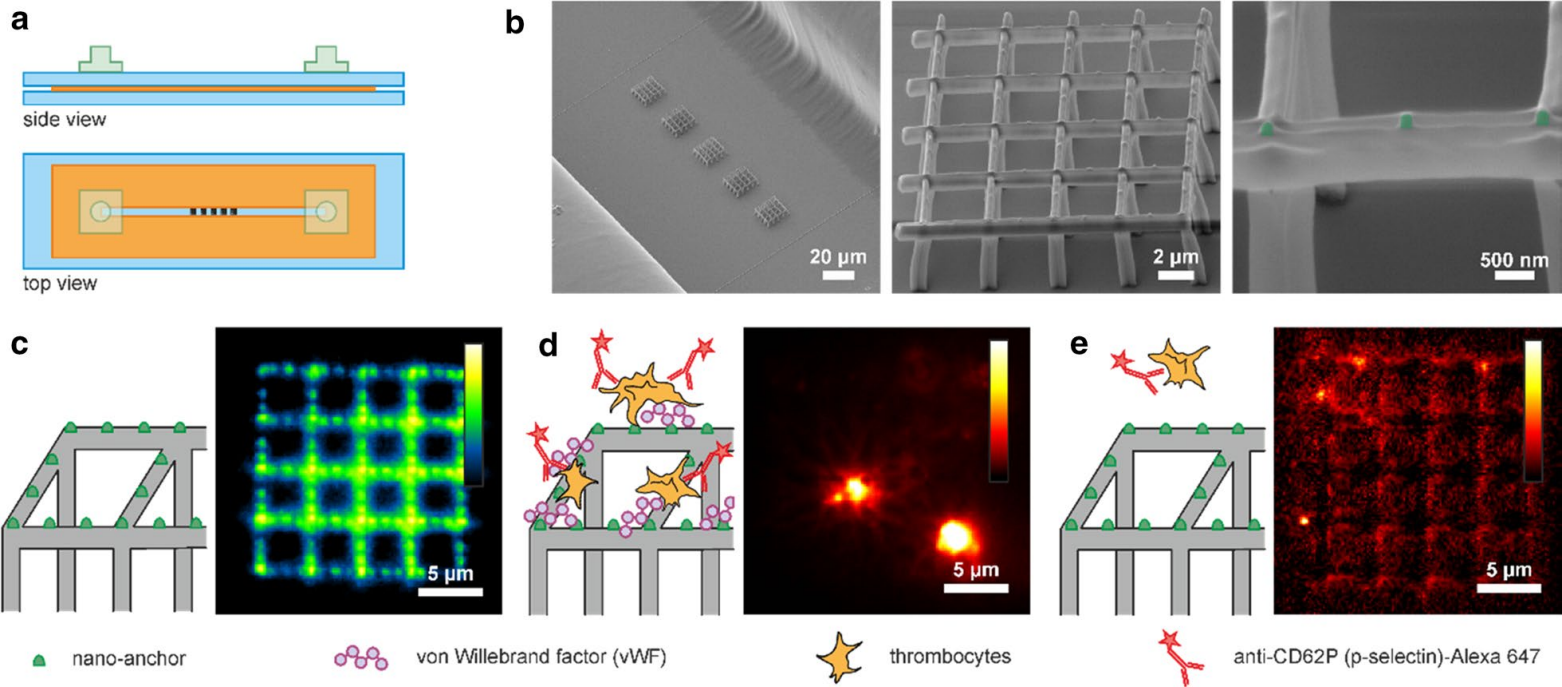
Microfluidic device for thrombocyte activation studies. **a** Sketch representing side- and top-view of the microfluidic device. Blue: glass slides, orange: double-sided adhesive tape with a thickness of 83.1 µm, green: in- and outlet connectors. **b** Scanning electron microscopy (SEM) images of the structures inside the microfluidic channel. Protein repellent MPL grids on posts (grey) carry STED-lithography written nanoanchors (green). **c** Left: Sketch of the protein repellent structure (grey) with protein binding nanoanchors (green). Right: Fluorescence microscopy image of an empty scaffold. Excitation wavelength: 491 nm, illumination time 5 ms. **d** Left: Sketch of the experiment for thrombocyte activation. The protein binding nanoanchors were labeled with von Willebrand factor (vWF) molecules. Subsequently, thrombocytes were added to the microfluidic device. Fluorescently labeled antibodies (a-CD62P Alexa 647) were bound to the activated thrombocytes. Right: Fluorescence image of activated thrombocytes bound on top of the scaffolds. Excitation wavelength: 642 nm, illumination time 5 ms. **e** Left: Sketch of the control experiment where vWF molecules were omitted. The structures were incubated with a thrombocyte concentrate under flow conditions and subsequently incubated with fluorescently labeled a-CD62p antibodies. Right: There were no activated thrombocytes present on the structures. Excitation wavelength: 642 nm, illumination time 5 ms

A microfluidic device (Fig. 1a) for thrombocyte activation studies comprises two glass slides (# 1.5, Menzel Gläser, Germany) stuck together with double-sided adhesive tape with a thickness of 81.3 µm (ARcare^®^ 90,445 Adhesives Research, Ireland). Channels of 20 mm in length with a width of ~ 190 µm were cut into the adhesive tape. Two holes were drilled in the top glass slide and fluidic in- and outlet connectors (kindly provided by EV Group, Austria) were attached using double-sided adhesive tape.

Before constructing the flow channel, three-dimensional structures were written on the bottom glass slide. More information on preparation of the glass slides can be found in the Supporting Information. A detailed description of the lithography setup has been previously published in reference [46]. In brief, a 780 nm pulsed laser (100 fs pulse duration, 50 MHz repetition rate, FemtoRay 780, Menlo Systems, Germany) was used for fabrication of the 3D polymer grids by focusing the beam into a photoresist using an objective lens with a high numerical aperture (NA = 1.46). The protein repellent carrier scaffolds comprise grids of 5 × 5 bars on top of 5 µm high posts (Fig. 1b). The side length of the grids is 16 × 16 µm. The grids on posts were fabricated using a 4:1 mixture of the monomers pentaerythritol triacrylate (PETA, Sigma Aldrich, USA) and poly(ethylene glycol) diacrylate (PEG-DA, Sigma Aldrich, USA) with 1 wt.% Irgacure^®^ 819 (BASF, Switzerland) as photoinitiator. The chemical structures of the photoresist ingredients can be found in Additional file 1: Figure S1. Typically, excitation powers of 6.5–9 mW (measured prior to entering the objective lens) and writing speeds of 10–20 µm/s were used for structuring. Multiple exposures were applied to increase the mechanical strength and stability of the scaffolds.

Subsequently, nanoanchors for protein binding were attached on top of the grids using STED lithography. STED lithography was carried out using the same femtosecond laser used for MPL lithography and in addition, a second laser beam (532 nm, continuous wave, VerdiV5, Coherent, USA) with a point spread function shaped as a donut. This time, the photoresist contained a 9:1 mixture of PETA and 2-carboxyethyl acrylate (CEA, Sigma Aldrich, USA) with 0.25 wt.% of the photoinitiator 7-diethylamino-3-thenoylcoumarin (DETC, Acros Organics, Belgium) which is typically used in STED lithography [8, 9]. An excitation power of 3.5 mW, an illumination time of 5 ms and a depletion power of 18 mW were used for writing nanoanchors with lateral feature sizes of ~ 65 nm and a height of ~ 200 nm. Finally, the structures were developed using acetone. In a next step, the adhesive tape with the flow channel cut out was carefully placed on top such that the grids were aligned inside the flow channel (Fig. 1b) and the top glass slide was mounted to close the channels (Fig. 1a). Each channel contained five structures with a distance of 20 µm. The density of nanoanchors on the first four structures along the direction of flow was adjusted, to 9, 25 and 65 nanoanchors per grid, which we call low, medium, and high density of nanoanchors, respectively. The fifth structure, which we call the ‘readout’ structure, carried 145 nanoanchors in all the microfluidic experiments. Figure 1c shows a sketch of the structure with the protein-binding nanoanchors, which are visible in the green channel of a fluorescence microscope due to the DETC autofluorescence (excitation wavelength 488 nm). The grids and posts show negligible autofluorescence [43, 47].

### Thrombocyte activation studies

4-(2-hydroxyethyl)-1-piperazineethanesulfonic acid (HEPES) buffer (pH 7.5, 50 mM) was instilled into the microfluidic channel. Next, 100 µl vWF [48] diluted in HEPES buffer (concentration 10 µg/ml) was instilled and the structures were incubated for 20 min. After rinsing with HEPES buffer, 50 µl of a thrombocyte concentrate (diluted with 450 µl of a DMEM and PSFG mixture down to a concentration of ${2.9} \times {{10}}^{{3}}$ thrombocytes/µl) was infused and the structures were incubated for 25 min. The thrombocyte concentrate was kindly provided by the blood transfusion service; Linz, Austria. After another washing step with HEPES buffer, anti-CD62p (p-selectin) labeled with Alexa^®^ 647 (BioLegend, USA) was added (concentration 1 µg/ml) and the structures were incubated for 15 min. Figure 1d shows the structure with the activated and fluorescently labeled thrombocytes (excitation wavelength 642 nm, illumination time 5 ms). In the control experiments (Fig. 1e), the vWF was omitted and no thrombocytes were bound to the polymer scaffolds. Additional experiments on glass substrates directly structured with nanoanchors revealed that thrombocytes do not specifically activate on the nanoanchor-structured substrates (Additional file 1: Figure S2). The same nanoanchor densities were chosen as in the experiments with 3D scaffolds. In case of the absence of vWF, the densities of activated thrombocytes on the nanostructures and the surrounding glass were similar.

### Estimation of the number of vWF molecules on nanoanchors

The microfluidic channels with grids carrying the nanoanchors were flushed with HEPES buffer. Subsequently, 100 µl vWF diluted in HEPES buffer (concentration 10 µg/ml) was added for incubation for 20 min. After a washing step with HEPES buffer, 10 µl of 0.1 wt.% ovalbumin (albumin from chicken egg white, Sigma Aldrich, USA) in HEPES buffer was used for passivation to prevent nonspecific binding of the fluorescently labeled antibodies. After 10 minutes incubation and subsequent washing with HEPES buffer, 1 µl (concentration 1 µg/ml) of monoclonal mouse IgG antibodies F8/86 targeting vWF, labeled with Alexa^®^647 (Santa Cruz Biotechnology, USA), was added.

To quantify the number of vWF molecules attached to the nanoanchors, we used a statistical analysis of the fluorescence intensity per fluorescing spot from microscopy images [42]. An illumination time of 5 ms was used for all experiments. The signal of the labeled antibodies bound to vWF molecules attached to individual nanoanchors was compared to the signal of sparsely distributed antibodies attached to piranha-cleaned glass slides. The fitting algorithm is described in more detail in a study by Wiesbauer et al. [42]. Briefly, the intensity distribution of single IgG antibodies labeled with Alexa^®^647 was used as a reference. This reference distribution served as a weighted fit of the intensity distribution of the vWF attached to nanoanchors. From the weighting prefactors w_n_, one can then determine the number of antibodies per nanoanchor, which roughly corresponds to the number of immobilized vWFs. To determine the weighting prefactors w_n,_ the intensity distribution of single fluorescent IgG antibodies was analyzed and de-convolved with the intensity distribution of vWF molecules attached to the structures labeled with the same antibody (for more detail see [42]). Due to multiple anchored vWFs and the possibility that multiple antibodies could bind to individual vWFs, multiple weighting prefactors w_n_ were determined.

## Results and discussion

**Fig. 2:**
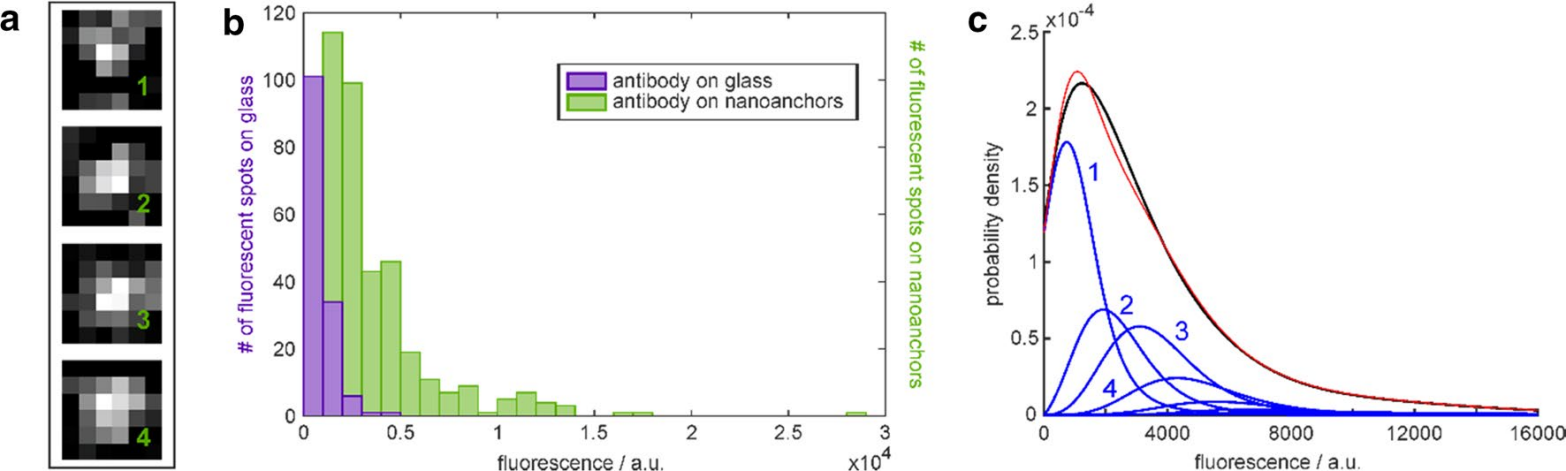
Estimation of the number of vWF molecules on nanoanchors. **a** Examples of fluorescence peaks on nanoanchors carrying most probably (from top to bottom) one (809 counts), two (1527 counts), three (2563 counts), and four (3306 counts) fluorescent IgG antibodies. **b** Distribution of Alexa647 fluorescence signals (a.u.) from anti-vWF IgGs. Purple: single antibody signals originating from sparsely and randomly distributed antibodies on a piranha-cleaned glass surface; green: fluorescence signals from nanoanchors loaded with vWF. Illumination time: 5 ms. **c** Black line: Probability density function fitted to the experimental signals from anti-vWF IgGs on the nanoanchors. The blue curve 1 is a weighted fit of the measured probability density function for sparsely distributed IgGs on glass and blue curves 2, 3, 4 etc. are the weighted extrapolations for 2, 3, 4 etc. IgGs, respectively. The red curve is the sum of the blue curves. The weighting indicates that 34% of the nanoanchors carry exactly one antibody, 20% carry two, 21% carry three, 10% carry four and 15% carry five or more fluorescent IgG antibodies

Figure 2a shows representative fluorescence signals of nanoanchors incubated with vWF and Alexa 647 labelled anti vWF IgGs. The nanoanchors carry most probably one (809 counts), two (1527 counts), three (2562 counts) or four (3306 counts) fluorescing IgG antibodies. Figure 2b depicts the intensity histograms of IgG antibodies labeled with Alexa^®^647 on glass (purple) and those of the IgG antibodies bound to the vWF molecules on nanoanchors (green). The median of the antibodies on glass (purple) is at 792 ± 48 counts, and the median of the antibodies on nanoanchors is at 2405 ± 145 counts (t_ill_ = 5 ms). This indicates that on average, three IgG are immobilized per nanoanchor, which also gives a rough estimate that there are approximately three vWF molecules per nanoanchor. In control experiments, vWF was omitted and the nanoanchors were passivated using ovalbumin. No IgG antibodies were bound to the nanoanchors. To compare and quantify the similarity of two distributions (namely, the fluorescence distribution of labeled antibodies bound to structures and the distribution of sparsely distributed antibodies on glass slides), a probability density fit algorithm, which estimates the average number of fluorescing anti-vWF antibodies per patch, was applied [49, 50]. Figure 2c shows the already weighted probability density distribution of anti-vWF labeled with Alexa^®^647 bound to vWF molecules. The weighted intensity distributions (blue lines) are weighted in such a way, that the sum of them (red line) fits best the measured probability density distribution (black line). It is retrieved that 34% of the vWF loaded anchors carry exactly one, 20% carry two, 21% carry three,10% carry four and 15% carry five or more fluorescent antibodies. Clearly, such low numbers of vWF molecules yielding a well-controlled vWF surface density can only be reached with STED-lithography, but not with MPL [42].

**Fig. 3:**
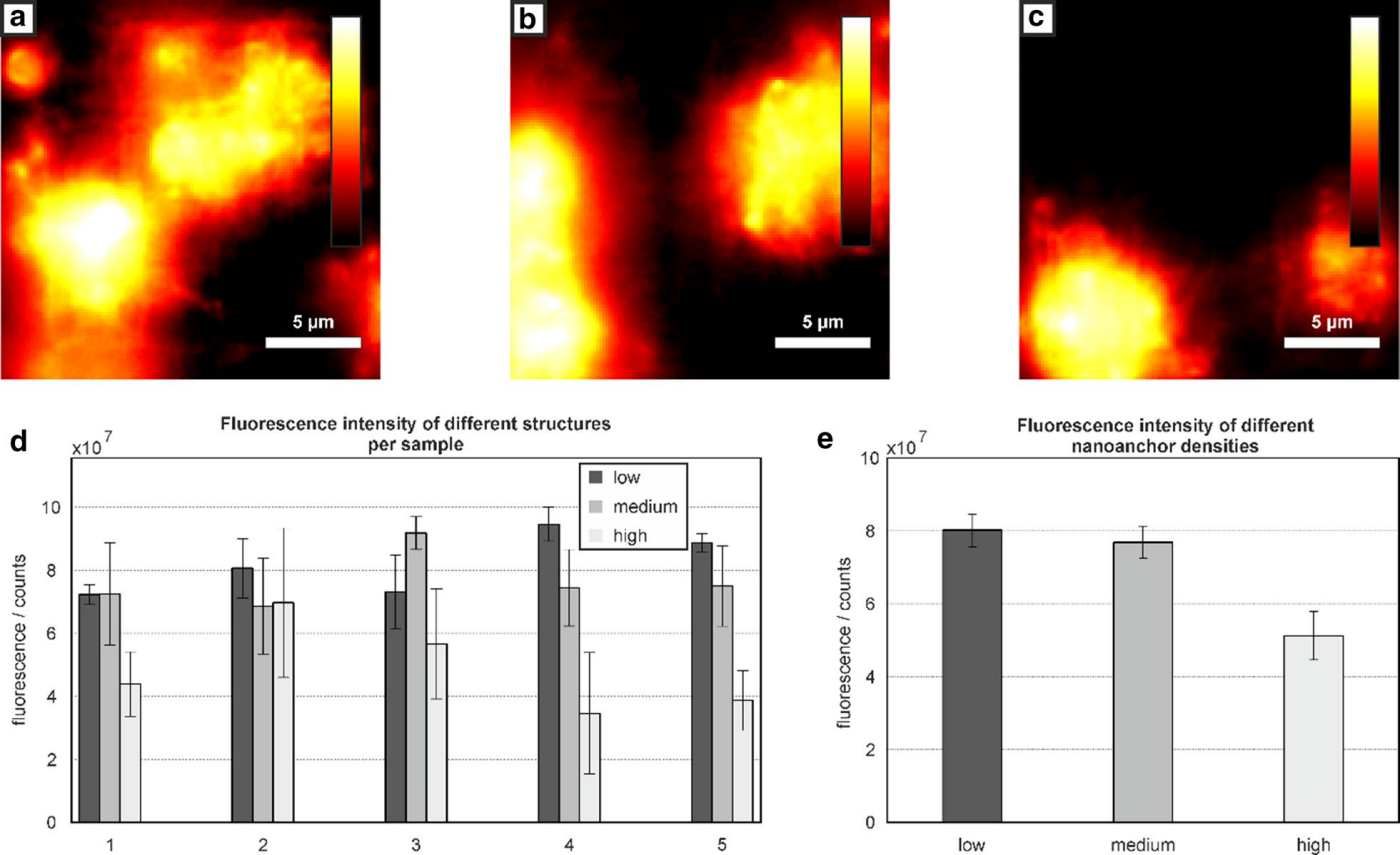
Thrombocyte activation studies. **a**–**c** Fluorescence images of activated thrombocytes on top of the 3D structures inside the microfluidic channels. The grids in **a**–**c** carry a low, medium and high density of nanoanchors with integrated intensities of ${5.17} \times {{10}}^{{7}}$ counts, ${4.34} \times {{10}}^{{7}}$ counts and ${2.54} \times {{10}}^{{7}}$ counts, respectively. **d** Fluorescence signal of anti-CD62p-Alexa^®^647, integrated in the proximity to each structure (picture section of 26.4 × 26.4 µm) for grids 1 to 5 in downstream direction. Each data point is averaged over four experiments. **e** Average fluorescence from the first four grids for the three experiments with low, medium and high density of nanoanchors

Figure 3a–c shows typical fluorescence images of three structures inside a microfluidic channel (t_ill_ = 5 ms). The number of binding sites on the grids shown in Fig. 3a–c are 9, 25 and 65 nanoanchors (low, medium and high nanoanchor density, respectively). Under the assumption that three vWF molecules are bound per nanoanchor, 27, 225 and 585 vWF molecules were estimated for the three different densities. While the first four structures in every channel carry either 9, 25 or 65 nanoanchors, the fifth grid always carries 145 nanoanchors and acts as a reference and quality check for every experiment

For quantification of thrombocyte activation, the signal of the anti-CD62p-Alexa^®^647 labeled-thrombocytes was determined. Figure 3d shows the integrated signal, determined individually from each grid inside the microfluidic channel. For every image (123 × 123 pixels per image) a threshold intensity was determined. The threshold was chosen to be the average intensity from the grid. Then, the number of pixels with intensities above the threshold intensity were determined and the intensity of the area was summed. For each dot density, four replicas were investigated and Fig. 3d shows the fluorescence for each of the five downstream grids, averaged over the four measurements. The analysis reveals that the lowest anti-CD62p-Alexa^®^647 signals could be determined from thrombocytes activated on the structures with the highest vWF density (65 nanoanchors/scaffold = 195 vWF proteins), compared to signals determined on structures with lower dot densities (18, 75 vWF molecules). Figure 3e depicts the average signal from 12 microfluidic devices (48 scaffolds) representing the three experimental setups. Only the first four scaffolds with low, medium and high densities of nanoanchors were considered for analysis. Interestingly, structures with higher densities of nanoanchors showed lower fluorescence signals than structures with a lower nanoanchor density, even for the downstream control grid. The physiological interpretation of this finding is beyond the scope of the current work.

## Conclusions

In this study, we developed a microfluidic chip containing MPL structures decorated with STED lithographically-written nanoanchors. We showed the applicability of STED enhanced three-dimensional structures within microfluidic channels for cell manipulation. The three-dimensional structure consists of a protein repellent MPL written scaffold which was decorated with STED-written nanoanchors, capable of vWF binding. Single molecule fluorescence microscopy enabled us to show that on average three IgG antibodies and thus approximately three vWF molecules were bound per nanoanchor.

STED-lithography allows for an assay with a high dynamic range. Writing of polymeric functional structures with feature sizes of ca. 65 nm allowed a tight control of the capacity of these nanoanchors. It gives a higher flexibility for spatial arrangement of single vWF molecules in 3D space. The impact of locally arranged low concentrations of vWF on thrombocytes and their activation in 3D under flow conditions was studied for the first time. Our platform shows the capability to perform such studies. Additionally, the optimized polymers allow fluorescence imaging at a single molecule level.

Real-time monitoring of the interactions of cells with artificially introduced 3D protein coated obstacles inside microfluidic devices can provide insights into both normal and abnormal cell behavior caused by the physical and chemical mechanisms of shear mediated cell-scaffold-interactions. Upon incubation with thrombocytes, we were able to show that 3D grids carrying nanoanchors with vWF molecules have an impact on activation. High nanoanchor densities lead to lower fluorescence signals from the thrombocytes in comparison to those exposed to low nanoanchor densities.

The microfluidic system presented in this work can further be used to test for multiple types of perturbations of thrombocyte activation, including those that are of a genetic nature as well as those that are drug induced. Our system combines not only a biochemical, but also a mechanical stimulation of the cells under flow conditions. Studies to elucidate the activation of platelets by means of density of functional ligands in combination with mechanical stress are of high interest in the thrombogenesis of blood and this platform might enable the study of pathological mechanisms related to slow or low trigger events in thrombogenesis as well as impairment of platelet function by drugs. Further extensions of this platform that can provide needed biological complexity for the system would involve modifying the materials used for structuring and the introduction of orthogonal polymer chemistry to the anchors, which would allow binding of multiple, different proteins (crucial for thrombocyte activation) [43, 46].

## Supplementary Information

**Additional file 1: Figure S1.** Chemical structures of the photoresist ingredients. **Figure S2** Thrombocyte activation on two-dimensional surfaces.

## Data Availability

The datasets used and/or analyzed during the current study are available from the corresponding author on reasonable request.

## Abbreviations

MPL: Multiphoton polymerization lithography
STED: stimulated emission depletion
vWF: Von Willebrand factor
PETA: pentaerythritol triacrylate
PEG-DA: poly(ethylene glycol) diacrylate
CEA: 2-carboxyethyl acrylate
DETC: 7-diethylamino-3-thenoylcoumarin
SEM: scanning electron microscopy
NA: numerical aperture
HEPES: 4-(2-hydroxyethyl)-1-piperazineethanesulfonic acid
