# Supplementary material for: STED lithography in microfluidics for 3D thrombocyte aggregation testing
Source: J Nanobiotechnology. 2021 Jan 18;19:23. doi: 10.1186/s12951-020-00762-8 (PMC7814651; doi:10.1186/s12951-020-00762-8)
Supplement: Supplementary file 1 — Additional file 1: Figure S1. Chemical structures of the photoresist ingredients. Figure S2 Thrombocyte activation on two-dimensional surfaces. [file 12951_2020_762_MOESM1_ESM.docx]

**Additional Information**

**STED Lithography in Microfluidics for 3D Thrombocytes Aggregation Testing**

Bianca Buchegger^a,b^, Alexander Tanzer^a^, Sandra Posch^c^, Christian Gabriel^d^, Thomas A. Klar^a^, Jaroslaw Jacak^b,^^[[1]](#footnote-1)^*

^a^ Institute of Applied Physics, Johannes Kepler University Linz, Altenberger Straße 69, 4040 Linz, Austria

^b^ University of Applied Sciences, Upper Austria School of Medical Engineering and Applied Social Sciences, Garnisonstraße 21, 4020 Linz, Austria

^c^ Department of Applied Experimental Biophysics, Institute of Biophysics, Johannes Kepler University Linz, Gruberstraße 40, 4020 Linz, Austria

^d^ Ludwig Boltzmann Institute for Experimental and Clinical Traumatology, Donaueschingenstraße 13, 1200 Vienna, Austria

**Preparation of glass coverslips**

Prior to fabrication of the polymer structures, the glass slides were treated as follows: First, they were cleaned with peroxymonosulfuric acid. Afterwards, the glass slides were dried in a compartment dryer at 150°C for 15 minutes. For better adhesion of the acrylate structures, the glass slides were, in a second step, put in a 1 mM solution of 3-(Trimethoxysilyl)propyl methacrylate (Sigma Aldrich, USA) in toluene for one hour. The glass cover slips were thoroughly rinsed with toluene and dried with nitrogen before being placed in a compartment dryer at 120°C for 10 minutes.

**Polymer Structures**

The three dimensional, protein repellent scaffolds were fabricated using a photoresist (Figure S1) with 1 wt.% Irgacure819 (BASF, Switzerland) as photoinitiator and two monomers: pentaerythritol triacrylate (PETA, Sigma Aldrich, USA) and poly(ethylene glycol) diacrylate (PEG-DA, Sigma Aldrich, USA) with a ratio of 4:1. The scaffolds carry nanometer sized binding pins – nanoanchors – fabricated using STED lithography. The protein adhesive photoresist (Figure S1) used contains PETA and 2-carboxyethyl acrylate (CEA, Sigma Aldrich, USA) (9:1) and 0.25 wt.% 7-diethylamino-thenoylcoumarine (DECT, Acros Organics, Belgium).


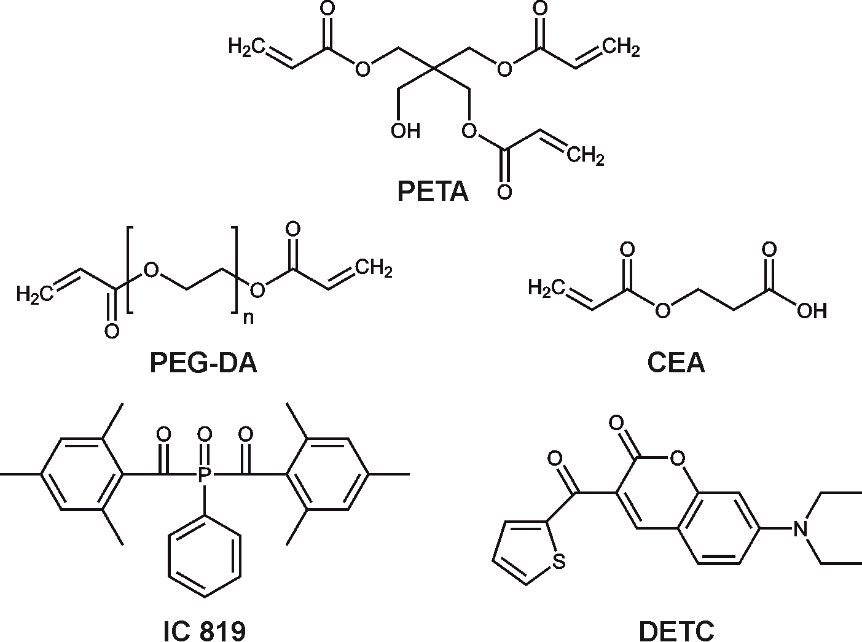


**Figure S1: Chemical structures of the photoresist ingredients.** The protein repellent photoresist for the three-dimensional scaffolds is a 4:1 mixture of pentaerythritol triacrylate and poly(ethylene glycol) diacrylate (PEG-DA) with 1 wt.% Irgacure 819 (IC 819). The protein adhesive photoresist for the STED written nanoanchors is 9:1 mixture of PETA and carboxyethyl acrylate (CEA) with 0.25 wt.% 7-diethylamino-3-thenoyl coumarine (DETC).

**2D experiments**

Additional experiments with two dimensional nanoanchor arrays within microfluidic channels were performed. The nanoanchors were fabricated directly at the substrate surface; the arrays of the nanoanchors as well as the spatial sequence of the nanoanchor arrays were similar to the three dimensional structures described in the manuscript (see Figure 1). The experimental protocol was similar to the experiments on thrombocyte activation on three dimensional structures. The results are depicted in Figure S2. Comparison of the images obtained from thrombocytes activated at the site of the nanoanchor arrays to images taken randomly at the substrate surface show activation of thrombocytes on the glass surface as well as on the nanoanchors. This motivates the use of three dimensional structures to increase the signal-to-noise ratio.


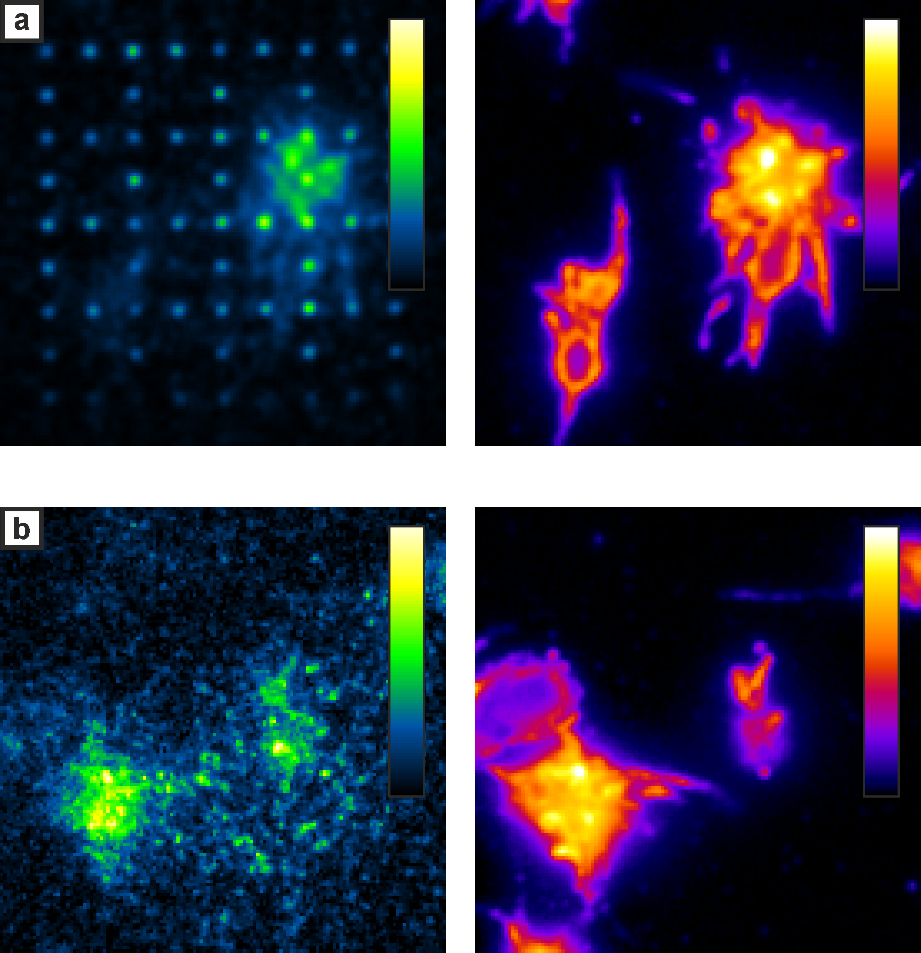


**Figure S2: Thrombocyte activation on two-dimensional surfaces.** a) Fluorescence images of the nanoanchors located at the glass substrate (left; excitation wavelength 491 nm) and activated thrombocytes labeled with anti-CD62p antibody conjugated to Alexa®647 fluorophore (right; excitation wavelength 642 nm). b) Fluorescence images of the glass surface (left) and activated thrombocytes (right) on the same sample but taken at a position without nanoanchors. The illumination time was set to 5 ms for every image.

1. * Corresponding Author: J. Jacak (jaroslaw.jacak@fh-linz.at) [↑](#footnote-ref-1)
